# Changes in the prevalence of metabolic syndrome in Korean adults after the COVID-19 outbreak

**DOI:** 10.4178/epih.e2022101

**Published:** 2022-11-05

**Authors:** Ji-Young Kwon, Sang-Wook Song

**Affiliations:** Department of Family Medicine, St. Vincent’s Hospital, College of Medicine, The Catholic University of Korea, Suwon, Korea

**Keywords:** COVID-19, Metabolic syndrome, Social isolation, Pandemics, SARS-CoV-2, Coronavirus

## Abstract

**OBJECTIVES:**

This study sought to reveal changes in the prevalence of metabolic syndrome (MetS) after the start of the coronavirus disease 2019 (COVID-19) pandemic and to identify the groups showing the greatest changes.

**METHODS:**

We compared the prevalence of MetS between 2017–2019 (i.e., the 3-year period before the COVID-19 pandemic) and soon after the initial outbreak of COVID-19 in 2020 among 24,564 adults ≥19 years of age using data from the Korea National Health and Nutrition Examination Survey.

**RESULTS:**

The prevalence of MetS increased steadily between 2017 and 2020 (29.4 to 35.3%, p for trend <0.001), with annual percent changes of 2.0%p, 2.2%p, and 1.7%p, respectively. Compared to 3 years before the COVID-19 pandemic, the prevalence of MetS significantly increased during the COVID-19 pandemic in males (6.2%p; 95% confidence interval [CI], 3.5 to 8.9) compared to females (1.5%p; 95% CI, −1.2 to 4.1). The age groups with the greatest increases in MetS prevalence after the COVID-19 pandemic were those in their 40s (4.6%p; 95% CI, 0.9 to 8.4) and 50s (5.8%p; 95% CI, 2.2 to 9.4). By educational background, the increase in MetS prevalence was greatest among those with a college degree or higher (5.1%p). The prevalence of MetS in high-income (5.3%p) and low-income (4.6%p) groups significantly increased.

**CONCLUSIONS:**

The increasing trend in the overall prevalence of MetS continued during the COVID-19 pandemic. In particular, the prevalence of MetS among adult males in their 40s and 50s increased significantly after the COVID-19 outbreak in Korea.

## GRAPHICAL ABSTRACT

[Fig f3-epih-44-e2022101]

## INTRODUCTION

Severe acute respiratory syndrome coronavirus 2 (SARS-CoV-2), capable of causing coronavirus disease 2019 (COVID-19), was first identified in December 2019 but quickly spread worldwide [1], and the World Health Organization (WHO) eventually declared a global pandemic on March 11, 2020 [2]. In the Korea, after the first confirmed case of infection in January 2020, a major epidemic began. Since then, the cumulative number of confirmed COVID-19 cases in Korea exceeded 18,680,000 in July 2022, and >24,700 deaths have been reported [3]. In Korea, varying crisis levels (from 1 to 4) for COVID-19 were implemented, with different degrees of intensity for lockdown orders that encouraged or mandated “social distancing” [4]. The Korean government adjusted the crisis level according to the regional severity of the spread of the disease. Through these response measures, the number of participants in private meetings was limited, access to public facilities became difficult, and nighttime operations were suspended. Students were also unable to attend school and had to study at home via online video classes [5].

The restrictions imposed by the COVID-19 pandemic have fundamentally changed the way people live, affecting their health and behaviors alike [6,7]. Restrictions on outside activities have forced people to stay at home for longer periods of time and increase their sedentary time [8]. An analysis of the Community Health Survey collected by the Korea Disease Control and Prevention Agency in 2020 reported a decrease of 49.6% in physical activity after the spread of SARS-CoV-2 [5]. Screen time, such as time spent watching television, playing online games, and using smartphones, has also increased [9]. Dietary behaviors have also trended negatively, with increased consumption of delivered food, fast food, and snacks [10,11]. These behavioral changes have led to an increased intake of high-calorie and high-fat foods. According to a study by the Korean Society for Obesity, 46% of Koreans gained >3 kg in weight during the COVID-19 pandemic due to reduced physical activity and increased unhealthy irregular eating habits [12]. Negative impacts were also found on mental health. Anxiety, depression, and stress about an uncertain future were exacerbated by prolonged lockdown periods and the contraction of economic activity [13–17]. This deterioration of mental health negatively affected physical activity and eating behaviors, further exacerbating the vicious circle.

Lifestyle changes since the COVID-19 lockdown have increased the prevalence of chronic diseases and obesity [8,18–20]. In a study conducted using Korea National Health and Nutrition Examination Survey (KNHANES) data, the prevalence of obesity, hypertension, diabetes, and hyperlipidemia increased by 6%p, 1.8%p, 1.9%p, and 2.8%p in males during the COVID-19 pandemic, respectively [18]. A study in Italy showed that the prevalence of obesity increased from 37.8% to 51.3% and that of metabolic syndrome (MetS) increased from 14.9% to 27.0% after the initial outbreak of COVID-19 [8]. MetS is a set of risk factors for cardiovascular disease associated with insulin resistance, including abdominal obesity, dyslipidemia, diabetes, and high blood pressure [21]. The prevalence of MetS is on the rise, being mainly associated with obesity and an increase in the number of individuals with sedentary lifestyles [6]. MetS is independently associated with the risk of cardiovascular disease and with cardiovascular mortality [22–26]. Reports have stated that MetS doubled the risk of cardiovascular disease and its associated mortality and stroke [27]. In addition, the presence of MetS was associated with the severity of COVID-19; specifically, a higher number of metabolic components increased the risk of severe COVID-19 linearly [19,28].

In Korea, a study using large-scale national data investigated changes in the prevalence of MetS up to 2018. However, no studies have compared its prevalence before and after the declaration of the COVID-19 pandemic. The purpose of this study was to investigate the impact of COVID-19 on the prevalence of MetS. In addition, we investigated the difference and degree of change in the prevalence of MetS according to demographic characteristics.

## MATERIALS AND METHODS

### Study population

We used data from KNHANES from 2017 to 2020. The KNHANES is a government-designated statistical investigation of the health behaviors of the national population, the prevalence of chronic diseases, and the status of food and nutrition intake in Korea [29]. The subjects of the survey were about 192 survey districts and 23–25 sample households per survey district every year, and all individuals ≥1 year of age were targeted. However, the 2020 survey was suspended as a result of the COVID-19 outbreak, and only 180 survey districts for health surveys and screening surveys and 166 survey districts for nutrition surveys completed their respective surveys. This study included only data from adults ≥19 years of age among the KNHANES data from 2017 to 2020. We compared 2017–2019 as the pre-pandemic period with 2020 as the pandemic period. Pregnant participants were excluded because pregnancy could affect waist circumference regardless of MetS.

### Data collection and study variables

The KNHANES investigations are carried out by a specialized investigation team affiliated with the Korea Disease Control and Prevention Agency in mobile examination vehicles. The height and weight of each participant are measured to 1 decimal place using a stadiometer and digital scale. For body mass index (BMI), weight (kg) divided by the square of height (m^2^) was used. Waist circumference was measured at the mid-point between the lower end of the last rib and the upper end of the iliac crest on the mid-axillary line while the subject was exhaling. Blood pressure was measured 3 times, and the average of the second and third measurements was automatically calculated and recorded. Blood samples were taken and analyzed from participants in fasting conditions for ≥8 hours.

To discern health behaviors, health questionnaire data on drinking, smoking, and physical activity were used. Current smokers were defined as those who had smoked ≥100 cigarettes in their lifetime and still smoked, and current drinkers were defined as those who had drank ≥1 drink per month in the past year. Regarding physical activity, responses were divided into whether or not aerobic physical activity was practiced. Aerobic activity adherence referred to engaging in moderate-intensity physical activity for ≥2.5 hours or engaging in high-intensity physical activity for ≥1 hour and 15 minutes. If a mixture of moderate-intensity physical activity and high-intensity physical activity was performed, 1 minute of high-intensity activity was considered to be the same as 2 minutes of moderate-intensity activity. Diabetes, hypertension, and dyslipidemia were investigated for participants’ medical history and defined according to a self-reported doctor’s diagnosis, regardless of whether a participant took a related drug.

As demographic characteristics, age, sex, marital status, education level, average monthly personal gross income, and occupation were included. The household income group was defined by the value of the equivalized household income quartile. Marital status was divided into single and married, and the married group was further divided into 2 groups: those living with their spouses and those who were separated, widowed, or divorced.

### Definition of metabolic syndrome

We defined MetS according to the NCEP-ATP III (modified National Cholesterol Education Program Adult Treatment Panel III) criteria [30]. MetS was diagnosed when ≥3 of the following 5 criteria were met: (1) a waist circumference of ≥90 cm for males or ≥85 cm for females (following Korean-specific cut-offs for abdominal obesity defined by the Korean Society of Obesity [31]), (2) a triglyceride level of ≥150 mg/dL or the use of drugs for this reason; (3) a high-density lipoprotein (HDL) cholesterol level of <40 mg/dL for male or <50 mg/dL for female or taking medications for this reason; (4) a systolic blood pressure (SBP) of ≥130 mmHg, a diastolic blood pressure (DBP) of ≥85 mmHg, or taking anti-hypertensive medications; (5) a fasting blood sugar level of ≥100 mg/dL or taking anti-diabetic medications.

### Statistical analysis

All statistical analyses were performed using SAS version 9.4 (SAS Institute Inc., Cary, NC, USA). Changes in MetS prevalence were stratified by sex and age because the effects of the COVID-19 pandemic have been reported to vary according to sex and age. We also performed pre-specified subgroup analyses based on marital status, education level, household income, and occupation. The values for categorical variables were calculated using SAS (PROC SURVEYFREQ; SAS Institute Inc.), and the values for continuous variables were similarly calculated using SAS (PROC SURVEYMEANS; SAS Institute Inc.). If p-value <0.05, the difference was considered to be statistically significant.

### Ethics statement

This study was approved by the Institutional Review Board of the Catholic University of Korea, St. Vincent Hospital (IRB approval No. VC22ZISE0119).

## RESULTS

### General characteristics of subjects

The general characteristics of the KNHANES participants from 2017 to 2020 are described in Table 1. The male-to-female ratio of the participants was 1:1, and the average age was 47–48 years. Participants were found to be evenly distributed in each age group. Alcohol consumption was less (p=0.001) in 2020 than in 2017–2019. Current smoking habits and physical activity also decreased, but without statistical significance. The prevalence rates of diabetes mellitus, hypertension, and dyslipidemia increased to 9.4% (p=0.001), 21.4% (p=0.041), and 18.6% (p=0.001), respectively, after the outbreak of COVID-19. The average BMI was 24.0±0.1 kg/m^2^ in 2019, which increased to 24.3±0.1 kg/m^2^ in 2020 (p for trend<0.001). The average hemoglobin A1c was 5.63±0.02% in 2017, which increased gradually to 5.77±0.02% in 2020 (p for trend <0.001). Compared with 2017–2019, fasting glucose also showed an increase in 2020, without statistical significance (p= 0.085). The average total cholesterol level was 190.9±0.6 mg/dL in 2020, which decreased compared to 2017–2019 (p=0.019). The average LDL cholesterol level also decreased to 113.8±1.5mg/dL in 2020 compared to 2017–2019 (p=0.002). The prevalence of MetS increased steadily between 2017 and 2020 (from 29.4 to 35.3%, p for trend <0.001). The annual percent changes (APCs) in prevalence from 2017 to 2020 were 2.0%p, 2.2%p, and 1.7%p, respectively.

### Comparison of metabolic syndrome prevalence before and after the COVID-19 pandemic

**Figure 1:**
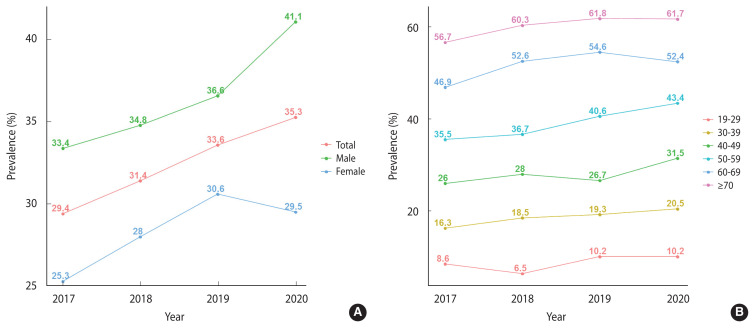
Trends in the prevalence of metabolic syndrome from 2017 to 2020 by sex (A) and age group (B).

Figure 1 shows the trends in prevalence according to sex and age groups and the APCs of MetS from 2017 to 2020. The prevalence of MetS steadily increased, and male showed a greater prevalence than female. The prevalence of MetS among male increased notably from 33.4% in 2017 to 41.1% in 2020. The APCs in these males were 1.4%p, 1.8%p, and 4.5%p, respectively, which significantly increased after the COVID-19 outbreak compared to before. In 2017, the prevalence among females was 25.3%, which increased by 2.7%p and 2.6%p every year thereafter. However, after the outbreak of COVID-19, it decreased by −1.1%p, reaching 29.5% in 2020. The prevalence of MetS increased with age each year. The groups with the largest difference in prevalence between 2019 and 2020 were those in their 40s and 50s, who experienced increases of 4.8%p and 2.8%p, respectively. However, the prevalence rates decreased in more advanced age groups (−2.2%p in those 60–69 years old and −0.1%p in those ≥70 years old).

**Figure 2:**
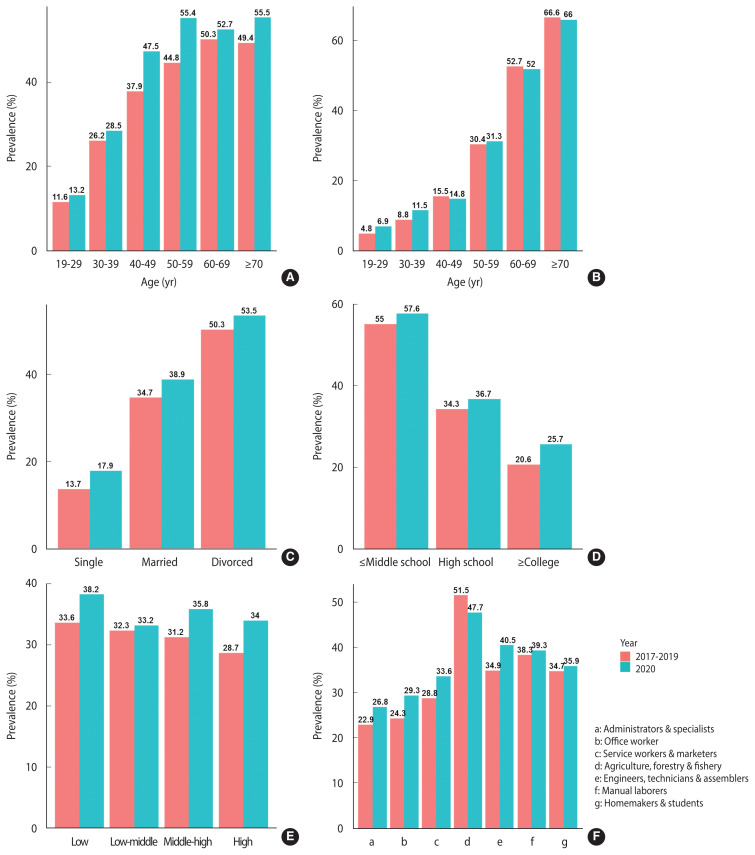
Differences in the prevalence of metabolic syndrome between 2017–2019 and 2020 according to age group in males (A), age group in females (B), marital status (C), education (D), household income (E), and occupation (F).

A comparison of the prevalence of MetS in the 3 years before the COVID-19 outbreak and in 2020 after the start of the COVID-19 pandemic is presented in Table 2, and Figure 2 shows a comparison of changes in the prevalence of MetS by age group for males and females, marital status, education level, income, and occupation. The overall prevalence difference was 3.8%p (95% CI, 1.6 to 6.0). The change in prevalence in males was 6.2%p (95% CI, 3.5 to 8.9), which was larger than that in females (1.5%p; 95% CI, −1.2 to 4.1), and the percentage change in females was not statistically significant. The age groups showing the greatest change in prevalence were those in their 40s and 50s, and there was a difference of 4.6%p (95% CI, 0.9 to 8.4) and 5.8%p (95% CI, 2.2 to 9.4), respectively. In males, the change in prevalence was greater in those 40–49 years old (9.6%p; 95% CI, 3.8 to 15.4) and 50–59 years old (10.6%p; 95% CI, 5.1 to 16.1). The prevalence also increased by 6.1%p (95% CI, 0.6 to 11.6) in those 70 years and older. In females, there were no statistically significant changes in prevalence according to age group.

When looking at the change in the prevalence of MetS after the outbreak of COVID-19 according to marital status, it was highest in both unmarried and married couples living together at 4.2%p (95% CI, 1.2 to 7.3 and 1.6 to 6.9, respectively). The prevalence was found to increase by 3.3%p in the separated, divorced, or widowed group, but the change was not statistically significant. The change in prevalence was 5.1%p (95% CI, 3.1 to 7.2) with the largest shift occurring in those who had graduated college or higher compared to those with less education. According to household income, the smallest change was found in the low-middle group (0.9%p; 95% CI, −2.6 to 4.4), and this change was not statistically significant. The change in prevalence was largest in the high-income group at 5.3%p (95% CI, 1.8 to 8.8), followed by the low-income group at 4.6%p (95% CI, 0.8 to 8.4). Among occupations, engineers, technicians, and assemblers showed the greatest change in prevalence, at 5.6%p (95% CI, 0.1 to 11.1).

## DISCUSSION

This study observed changes in the prevalence of MetS after social distancing measures were implemented due to the COVID-19 pandemic using large-scale Korean national data. From 2017 to 2020, the prevalence of MetS showed a steadily increasing trend. The prevalence of MetS in 2020 significantly increased compared to the average of the 3 years prior to the COVID-19 pandemic. In particular, the increase in prevalence was remarkable among males in their 40s and 50s.

According to a systematic review from 2017, in most countries, ≥20% of the adult population has been identified to have MetS, and this prevalence is gradually increasing [32]. In Korea, a fact sheet using KNHANES data from 2007 to 2018 was published [6], and the prevalence of MetS was almost constant from 2007 to 2014, then gradually began increasing after 2015. In this study, as in the previous study, the prevalence of MetS showed a tendency to increase. However, between 2019 and 2020—that is, before and after the COVID-19 outbreak—the overall APC did not significantly differ from previous APCs. This is because, between 2019 and 2020, the prevalence of MetS among males increased significantly by 4.5%p, while it decreased among females by 0.9%p.

When we compared the characteristics of the participants between 2017–2019 and 2020, the prevalence rates of diabetes, hypertension, and dyslipidemia also increased statistically significantly. In addition, the BMI was also observed to have increased. After the outbreak of COVID-19, these changes inevitably led to a rise in the prevalence of MetS, which was greatest in males in their 50s after the COVID-19 outbreak, followed by males in their 40s. The significant increase in the prevalence of MetS among males in their 40s and 50s appears to have been due to increased sedentary lifestyles, economic difficulties, and stress [6,18,29,33,34]. According to the Economically Active Population Census administered by the National Statistical Office of Korea [35], the labor force participation rate in 2020 was 77.9% for males versus 59.1% for females. In addition, in 2020 compared to 2019, the labor force participation rate decreased from 69.5% to 68.6%, and the unemployment rate increased from 3.8% to 4.0%. Males with a high labor force participation rate are more likely to have been exposed to psychological stress and the effects of increased sedentary lifestyles due to telecommuting. This would have made them more susceptible to dietary pattern changes and increased BMI values. A characteristic feature of the APC of MetS prevalence by age group during 2017–2020 is that the prevalence of MetS in the population aged 60 years, which had been on the rise, decreased after the COVID-19 outbreak. It seems that the younger generation suffered the most from the COVID-19 crisis. Younger people had more reduced levels of physical activity, were more likely to be stressed, and were more likely to drink excessively than older people. They were also more likely to eat delivery food as part of an unhealthy diet. After the COVID-19 pandemic, young people ≤40 years of age had negative changes in ≥1 health behaviors, while older people >70 years of age had positive changes [5]. The elderly population seems now to be more concerned about their health than before the outbreak of COVID-19 and made efforts to make positive changes to their lifestyle [34].

Unmarried people are younger and more likely to be economically active than those in other marital statuses. Depression in the COVID-19 era was confirmed to be more severe at younger ages [36], probably due to social isolation, loneliness, and uncertainty about the future. The relationship between depression and MetS has also been confirmed through a meta-analysis [37]. Those who are married and living together may have felt greater responsibility for their families due to the economic uncertainty and crisis following the COVID-19 outbreak. Furthermore, in some cases, as the whole family spends more time at home, conflicts within the family have increased. These changes increased stress and may have contributed to the increase in the prevalence of MetS. The change in the prevalence of MetS was greatest in those with a college degree or higher, probably because people with higher educational levels spend more time sitting and engage in less physical activity [5,38]. During the COVID-19 lockdown period, they may have been working sedentary, using computers for more hours than before. These would lead to unhealthy eating habits [5,39]. Most people with high incomes have a high level of education. Therefore, they also tend to lead a sedentary lifestyle because they are more likely to work at a desk. These sedentary lifestyles may have contributed to an increase in obesity and MetS prevalence [40]. In contrast, those with low income would have been most vulnerable to anxiety and stress as they were greatly exposed to economic uncertainty caused by rising unemployment, declining income, and financial difficulties [13,14].

There are several limitations to this study. First, as this study was not a longitudinal study of the same subjects, its findings do not provide definitive support for a causal relationship. Second, after the outbreak of COVID-19, Korea announced an active response policy from February 23, 2020, and since KNHANES data were steadily collected from January to December 2020, the periodical impact of social isolation may vary among subjects. Finally, since the KNHANES data in 2020 were collected within a short period of time after the outbreak of COVID-19, the increase in the prevalence of MetS cannot be confirmed to have been due to the impact of the COVID-19 lockdown. Furthermore, the available data to date are limited to 2020 and earlier, and there has been insufficient research into the impact of the COVID-19 lockdown period beyond 2021. However, this study is meaningful in that it observed changes in the prevalence of MetS in Koreans during the COVID-19 pandemic using objective measurement results obtained according to a standardized protocol using large-scale national data. This study also makes a meaningful contribution by providing information on groups with large changes in MetS prevalence after the COVID-19 outbreak.

In conclusion, the prevalence of MetS has increased steadily since 2017 and has continued to increase following the outbreak of COVID-19. In particular, efforts are needed to improve the lifestyle of males in their 40s and 50s, which is a group that experienced a significant increase in the prevalence of MetS. Even after 2021, as the COVID-19 pandemic continues and the period of social isolation becomes longer, it is necessary to continue observing trends in these changes.

## Figures and Tables

**Figure f3-epih-44-e2022101:**
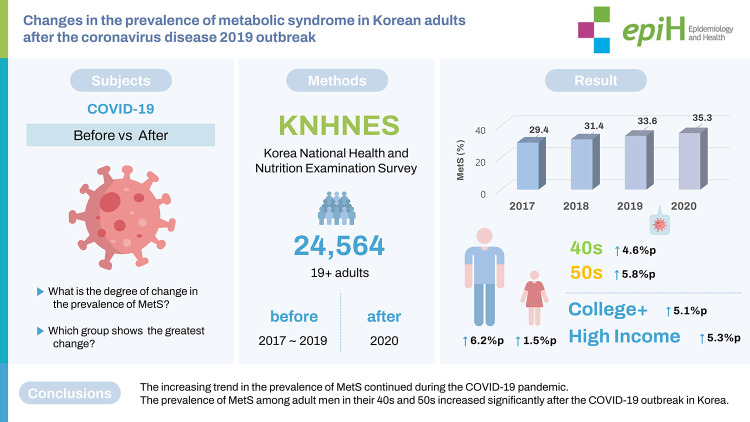


**Table 1.** General characteristics of the KNHANES participants from 2017 to 2020

Characteristics		Total (n=24,564)	2017 (n=6,167)	2018 (n=6,216)	2019 (n=6,269)	2020 (n=5,912)	p-value (2017-2019) vs. 2020	p for trend
Sex							0.696	0.800
	Male	10,931 (50.0)	2,749 (50.0)	2,736 (50.1)	2,795 (50.0)	2,651 (50.0)		
	Female	13,633 (50.0)	3,418 (50.0)	3,480 (49.9)	3,474 (50.0)	3,261 (50.0)		
Age (yr)		47.8±0.2	47.4±0.5	47.6±0.4	48.0±0.5	48.3±0.5	0.262	0.122
	19-29	3,030 (17.5)	724 (17.5)	759 (17.8)	749 (17.4)	798 (17.3)	0.558	0.118
	30-39	3,462 (17.0)	893 (17.6)	899 (17.2)	907 (16.8)	763 (16.5)		
	40-49	4,368 (19.7)	1,138 (20.5)	1,134 (20.0)	1,124 (19.4)	972 (19.1)		
	50-59	4,664 (20.0)	1,215 (20.0)	1,198 (19.9)	1,186 (20.0)	1,065 (19.8)		
	60-69	4,469 (13.9)	1,101 (13.0)	1,109 (13.5)	1,135 (14.2)	1,124 (14.9)		
	≥70	4,571 (12.0)	1,096 (11.4)	1,117 (11.8)	1,168 (12.2)	1,190 (12.6)		
Alcohol use, current							0.001	<0.001
	No	8,608 (36.9)	2,062 (34.6)	2,210 (36.9)	2,155 (36.5)	2,175 (39.3)		
	Yes	12,931 (63.1)	3,325 (65.4)	3,315 (63.1)	3,322 (63.5)	2,969 (60.7)		
Smoking, current							0.063	0.053
	No	19,972 (79.4)	4,995 (78.8)	5,029 (78.5)	5,081 (79.6)	4,867 (80.6)		
	Yes	4,289 (20.6)	1,094 (21.2)	1,132 (21.5)	1,082 (20.4)	981 (19.4)		
Aerobic activity adherence							0.156	0.133
	No	13,239 (54.7)	3,301 (53.5)	3,469 (55.1)	3,343 (54.3)	3,126 (56.0)		
	Yes	9,764 (45.3)	2,483 (46.5)	2,471 (44.9)	2,548 (45.7)	2,262 (44.0)		
Medical history							0.001	0.002
	DM	2,477 (8.1)	596 (7.5)	574 (7.3)	624 (8.2)	683 (9.4)		
	HBP	6,152 (20.0)	1,482 (18.5)	1,491 (19.2)	1,609 (20.8)	1,570 (21.4)	0.041	0.003
	Dyslipidemia	4,900 (16.8)	1,167 (16.2)	1,198 (15.7)	1,221 (16.5)	1,314 (18.6)	0.001	0.003
Marital status							0.169	0.426
	Single	4,385 (23.9)	1,037 (23.4)	1,096 (24.0)	1,079 (23.0)	1,173 (25.4)		
	Married (cohabitation)	16,555 (64.9)	4,236 (65.7)	4,220 (64.5)	4,283 (66.2)	3,816 (63.2)		
	Divorced/Widowed/Separated	3,608 (11.2)	890 (10.9)	897 (11.5)	905 (10.8)	916 (11.4)		
Education							0.054	0.040
	≤Middle school	6,211 (19.5)	1,723 (21.8)	1,620 (20.3)	1,540 (18.8)	1,328 (17.1)		
	High school	6,301 (27.7)	1,502 (25.7)	1,657 (28.4)	1,643 (28.2)	1,499 (28.6)		
	≥College	10,511 (52.7)	2,569 (52.4)	2,664 (51.4)	2,712 (53.0)	2,566 (54.3)		
Household income							0.841	0.466
	Low	6,108 (25.1)	1,535 (25.2)	1,547 (26.0)	1,565 (24.6)	1,461 (24.5)		
	Low-middle	6,135 (25.0)	1,549 (24.8)	1,557 (25.9)	1,556 (24.9)	1,473 (24.6)		
	Middle-high	6,098 (25.1)	1,512 (24.9)	1,550 (24.7)	1,564 (25.6)	1,472 (25.4)		
	High	6,116 (24.8)	1,546 (25.1)	1,541 (23.4)	1,550 (25.0)	1,479 (25.5)		
Occupation							0.571	0.228
	Administrators and specialists	3,316 (16.5)	848 (17.1)	895 (17.0)	809 (15.8)	764 (16.2)		
	Office worker	2,482 (12.3)	64 8(12.8)	611 (11.6)	668 (12.9)	555 (11.7)		
	Service workers and marketers	2,978 (13.9)	660 (12.1)	843 (15.4)	736 (13.7)	739 (14.4)		
	Agriculture, forestry, and fishery	869 (2.6)	279 (3.3)	228 (2.7)	175 (2.1)	187 (2.4)		
	Engineers, technicians, and assemblers	2,308 (11.6)	585 (11.9)	609 (12.1)	614 (11.9)	500 (10.6)		
	Manual laborers	2,052 (7.9)	501 (7.9)	506 (7.3)	554 (8.1)	491 (8.0)		
	Homemakers and students	8,974 (35.2)	2,271 (35.0)	2,238 (33.9)	2,320 (35.5)	2,145 (36.7)		
BMI (kg/m^2^)		24.0±0.0	23.9±0.1	24.0±0.1	24.0±0.1	24.3±0.1	<0.001	<0.001
Obesity (BMI ≥25 kg/m^2^)							<0.001	0.003
	No	15,757 (64.4)	4,004 (65.3)	4,029 (65.0)	4,110 (65.6)	3,614 (61.6)		
	Yes	8,615 (35.7)	2,148 (34.7)	2,142 (35.0)	2,123 (34.4)	2,202 (38.4)		
Waist circumference (cm)		83.1±0.1	81.7±0.2	82.2±0.2	83.8±0.2	84.7±0.2	<0.001	<0.001
Systolic BP (mmHg)		118.0±0.2	117.5±0.4	117.8±0.4	118.5±0.3	118.3±0.4	0.426	0.053
Diastolic BP (mmHg)		76.0±0.1	75.8±0.2	76.0±0.2	76.0±0.2	76.1±0.2	0.414	0.271
HbA1c (%)		5.70±0.01	5.63±0.02	5.65±0.01	5.75±0.01	5.77±0.02	<0.001	<0.001
Fasting glucose (mg/dL)		100.4±0.2	99.8±0.4	100.2±0.3	100.5±0.4	101.0±0.4	0.085	0.027
Total cholesterol (mg/dL)		192.1±0.3	193.2±0.7	191.7±0.7	192.8±0.6	190.9±0.6	0.019	0.039
Triglycerides (mg/dL)		135.1±0.9	135.7±1.8	136.2±1.8	131.8±1.7	136.7±2.2	0.368	0.900
LDL-cholesterol (mg/dL)		117.7±0.8	121.1±1.6	117.9±1.4	118.4±1.5	113.8±1.5	0.002	0.002
HDL-cholesterol (mg/dL)		51.6±0.1	51.1±0.2	51.0±0.3	52.7±0.3	51.4±0.2	0.536	0.005
Metabolic syndrome		8,910 (32.4)	2,069 (29.4)	2,185 (31.4)	2,316 (33.6)	2,340 (35.3)	0.001	<0.001

Values are presented as number (weighted %) or mean±standard error. KNEANES, Korea National Health and Nutritional Examination Survey; DM, diabetes mellitus; HBP, high blood pressure; BP, blood pressure, BMI, body mass index; LDL, low-density lipoprotein; HDL, high-density lipoprotein.

**Table 2.** Changes in the prevalence of metabolic syndrome in 2020 compared to 3 years before the COVID-19 pandemic

Variables		2017-2019	2020	Changes		
				Difference (%p)	95% CI	
					LL	UL
Total, age ≥19 (yr)		31.5 (30.5, 32.4)	35.3 (33.4, 37.1)	3.8	1.6	6.0
	19-29	8.4 (7.1, 9.8)	10.2 (8.0, 12.3)	1.7	-0.9	4.3
	30-39	18.0 (16.3, 19.7)	20.5 (16.8, 24.2)	2.5	-1.6	6.5
	40-49	26.9 (25.1, 28.7)	31.5 (28.2, 34.8)	4.6	0.9	8.4
	50-59	37.6 (35.8, 39.5)	43.4 (40.3, 46.4)	5.8	2.2	9.4
	60-69	51.5 (49.5, 53.5)	52.4 (48.1, 56.6)	0.9	-3.9	5.6
	≥70	59.7 (57.7, 61.6)	61.7 (58.5, 64.9)	2.1	-1.7	5.8
Male, age ≥19 (yr)		34.9 (33.7, 36.2)	41.1 (38.7, 43.5)	6.2	3.5	8.9
	19-29	11.6 (9.4, 13.9)	13.2 (9.6, 16.7)	1.5	-2.6	5.7
	30-39	26.2 (23.5, 28.8)	28.5 (23.0, 34.1)	2.4	-3.8	8.5
	40-49	37.9 (35.1, 40.7)	47.5 (42.4, 52.6)	9.6	3.8	15.4
	50-59	44.8 (42.1, 47.5)	55.4 (50.6, 60.1)	10.6	5.1	16.1
	60-69	50.3 (47.5, 53.0)	52.7 (46.7, 58.7)	2.5	-4.2	9.1
	≥70	49.4 (46.5, 52.4)	55.5 (50.8, 60.2)	6.1	0.6	11.6
Female, age ≥19 (yr)		28.0 (26.8, 29.2)	29.5 (27.2, 31.7)	1.5	-1.2	4.1
	19-29	4.8 (3.3, 6.2)	6.9 (4.2, 9.5)	2.1	-1.0	5.1
	30-39	8.8 (7.0, 10.5)	11.5 (7.7, 15.3)	2.7	-1.5	7.0
	40-49	15.5 (13.6, 17.4)	14.8 (11.3, 18.4)	-0.7	-4.7	3.3
	50-59	30.4 (28.1, 32.7)	31.3 (27.0, 35.7)	0.9	-4.1	5.9
	60-69	52.7 (50.0, 55.4)	52.0 (47.3, 56.7)	-0.7	-6.2	4.8
	≥70	66.6 (64.2, 68.9)	66.0 (61.8, 70.3)	-0.5	-5.4	4.3
Marital status						
	Single	13.7 (12.2, 15.2)	17.9 (15.3, 20.6)	4.2	1.2	7.3
	Married (cohabitation)	34.7 (33.5, 35.8)	38.9 (36.6, 41.2)	4.2	1.6	6.9
	Divorced/Widowed/Separated	50.3 (48.0, 52.5)	53.5 (49.6, 57.5)	3.3	-1.3	7.8
Education						
	≤Middle school	55.0 (53.4, 56.7)	57.6 (53.7, 61.4)	2.5	-1.7	6.8
	High school	34.3 (32.7, 35.9)	36.7 (33.4, 39.9)	2.3	-1.3	5.9
	≥College	20.6 (19.6, 21.6)	25.7 (23.9, 27.5)	5.1	3.1	7.2
Household income						
	Low	33.6 (31.9, 35.3)	38.2 (34.8, 41.5)	4.6	0.8	8.4
	Low-middle	32.3 (30.6, 34.0)	33.2 (30.2, 36.2)	0.9	-2.6	4.4
	Middle-high	31.2 (29.6, 32.8)	35.8 (32.8, 38.9)	4.6	1.2	8.1
	High	28.7 (27.0, 30.3)	34.0 (30.9, 37.0)	5.3	1.8	8.8
Occupation						
	Administrators and specialists	22.9 (21.2, 24.7)	26.8 (23.7, 29.9)	3.9	0.3	7.5
	Office worker	24.3 (22.0, 26.6)	29.3 (24.6, 33.9)	5.0	-0.3	10.2
	Service workers and marketers	28.8 (26.7, 31.0)	33.6 (29.3, 38.0)	4.8	0.0	9.7
	Agriculture, forestry, and fishery	51.5 (46.6, 56.5)	47.7 (34.7, 60.7)	-3.8	-17.8	10.1
	Engineers, technicians, and assemblers	34.9 (32.3, 37.5)	40.5 (35.6, 45.3)	5.6	0.1	11.1
	Manual laborers	38.3 (35.4, 41.3)	39.3 (34.3, 44.3)	1.0	-4.9	6.9
	Homemakers and students	34.7 (33.2, 36.3)	35.9 (33.3, 38.5)	1.2	-2.0	4.3

Values are presented as weighted % (95% CI). COVID-19, coronavirus disease 2019; CI, confidence interval; %p, percentage points; LL, lower limit; UL, upper limit.
